# Loneliness, Emotion Dysregulation, and Internalizing Symptoms During Coronavirus Disease 2019: A Structural Equation Modeling Approach

**DOI:** 10.3389/fpsyt.2020.581494

**Published:** 2021-01-08

**Authors:** Patrizia Velotti, Guyonne Rogier, Sara Beomonte Zobel, Rosetta Castellano, Renata Tambelli

**Affiliations:** Department of Dynamic and Clinical Psychology, Faculty of Medicine and Psychology, Sapienza University of Rome, Rome, Italy

**Keywords:** loneliness, emotion dysregulation, depression, stress, anxiety

## Abstract

**Background:** Our study aimed to test the hypotheses that an increased level of loneliness experienced during coronavirus disease 2019 (COVID-19) confinement was predictive of internalizing symptoms and that this pathway was mediated by emotion dysregulation levels.

**Methods:** To reach this aim, we performed an online longitudinal survey recruiting 1,330 participants at Time 1 (at the beginning of the lockdown) and 308 participants at Time 2 (few days before the end of the lockdown). All filled out a set of questionnaires: demographic data, University of California, Los Angeles Loneliness scale, Difficulties in Emotion Regulation Scale−18 items, and Depression Anxiety and Stress Scale−21 items. Hypotheses were tested using structural equation modeling in two steps and controlling for age. First, hypotheses were tested on cross-sectional data. Then, a cross-lagged panel analysis was performed on longitudinal data.

**Results:** Models obtained a good fit and evidenced the predictive role of loneliness levels on the three outcomes (i.e., depression, anxiety, and stress). Moreover, we found that emotion dysregulation levels partially mediated the longitudinal relationship between loneliness and both depression and stress but not between loneliness and anxiety levels.

**Conclusions:** This study points out that a central goal of clinical intervention could be the ability to regulate negative emotional states.

## Introduction

The coronavirus disease 2019 (COVID-19) pandemic poses challenges never before faced in society from many angles. It affects not only the state of health and integrity of our body endangered by COVID-19 but also the health and integrity of our mind, and the effects on the mental health of COVID-19 are expected to be diverse (1).

During a pandemic, not only the virus, with the consequent fear of contagion and death, but also the lockdown measures imposed by the various states contribute as stress factors affecting people's well-being (2–10). Indeed, according to a survey conducted in the United Kingdom on pandemic concerns (1), the prospect of contracting the severe acute respiratory syndrome coronavirus 2 was judged to be less worrying compared with psychological and social responses to the situation.

Furthermore, the answers of governments to fight the spreading of the virus, remaining physically distanced from relatives and friends, have determined a common condition of social isolation. As already known from the literature, social isolation is considered a risk factor for diverse psychopathological manifestations, such as anxiety (5, 6, 11), depressed mood (6, 7, 12, 13), substance abuse, domestic violence, suicide, and self-harm (14–16) and a trigger for some threatening conditions such as loneliness. A rapid review conducted by Brooks et al. (17) provided an overview of psychological effects related to quarantine, which occurred in acute, going from general psychological distress to post-traumatic stress symptoms (8, 18). Some studies have investigated long-term effects of the isolation experience with follow-ups, finding that PTSD and depressive symptoms persisted in a part of the sample even after 3 years from the epidemic outburst (13, 19).

Indeed, a wide body of research suggests that social isolation (i.e., the objective condition of isolation), psychological stress, and loneliness (i.e., subjective condition of isolation) have an unfavorable effect on many health outcomes, including mortality (20, 21). For this reason, the increase in social isolation and loneliness needs to be considered among the most important probable negative consequence of COVID-19, as evidenced by the surveys conducted by Holmes et al. (1).

## Loneliness and Emotion Regulation

Loneliness, defined as the pain of feeling alone (22), is a psychological condition characterized by a deep sense of emptiness and uselessness, lack of control, and personal threat (23, 24). Studies have shown that loneliness can lead to more serious physical and mental health problems such as internet addiction, suicide ideation, and substance use (25, 26). Loneliness also seems to be linked to internalizing symptoms such as depression in both adolescents (27–31) and adults (32–35), anxiety (36, 37), and—moreover—social anxiety (32, 38, 39).

However, research (40) highlights that diverse factors are involved in loneliness, such as individual (i.e., personality features) and contextual facets (i.e., social isolation). For instance, a meta-analysis study focused on adolescence found that the most powerful predictors of loneliness were individual characteristics such as low self-esteem and social anxiety (41). Instead, from a review that investigated the phenomenon in older adults, it came out that the psychological characteristics strongly linked to loneliness were cognitive deficits, poor mental health, negative life events, and low self-efficacy beliefs (42).

From an individual perspective, emotion regulation, defined as the complex set of psychic processes that translate into one's ability to influence his/her emotions, how he/she experiences them and how he/she expresses them (43), has a decisive role in promoting environmental adaptation and the following well-being (44). Literature has also highlighted the role of difficulties in emotion regulation as a risk factor for behavioral (45–47) and emotional problems such as substance abuse, gambling disorder, anxiety, and depression (48–51).

Nevertheless, individuals with the same level of loneliness may not experience similar psychological outcomes (52). This suggests that there might be mechanisms underlying the relationship between loneliness and internalizing symptoms, one of which seems to be emotion regulation difficulties. In fact, it has been shown that lonelier individuals make less use of adaptive emotional regulation strategies compared with individuals who suffer less from loneliness (53).

In light of these pieces of evidence, the main purpose of this study was to examine—through a structural equation model—the relationship between loneliness and internalizing symptoms, considering the mediating effect of difficulties in emotion regulation and the longitudinal link between loneliness and internalizing symptoms.

## Methods

### Participants and Procedure

For the purposes of the study, an online survey was created and diffused online 3 days after the beginning of the confinement in XXX. At the beginning of the survey, an exhaustive presentation of the study's aims and scopes was delivered and information concerning anonymity and privacy. Then, the participant was asked to sign an informed consent. Three days before the end of the national lockdown, participants were sent an email asking them to fulfill the same battery of self-report questionnaires. The procedure of the study applied with the American Psychological Association official guidelines and was approved by the Ethical Committee of the University of XXX, XXXX (N. 356/20).

### Measures

#### Demographic Information

For the purpose of the study, an initial questionnaire was created to evaluate information such as age, sex, and socioeconomic status.

#### University of California, Los Angeles Loneliness Scale

University of California, Los Angeles (UCLA) Loneliness Scale [(54), Italian version by (55) was used to measure the perceived level of loneliness. The instrument is a self-report questionnaire consisting of 20 items on a four-point Likert-type scale. Empirical literature evidenced the existence of three factors underlying the structure of the instrument, namely *Intimate Others* (intimate and interpersonal loneliness), *Social Others* (lack of social networks or social friendships), and *Affiliative Environment* (a lack of belonging to the affiliative environment). In the present study, the instrument showed good internal consistency with a Cronbach alpha coefficient of 0.82.

#### Difficulties in Emotion Regulation Scale 18 Items

Difficulties in Emotion Regulation Scale 18 items (56) is the short version of the Difficulties in Emotion Regulation Scale (DERS) (57, 58). As DERS, the instrument asks the participant to answer on a five-point Likert-type scale. The scale measures emotion dysregulation levels providing a total score and six separated scores related to the subscales of the instrument being *Non-Acceptance* (difficulty to accept in a non-judgmental way one's negative emotional states), *Awareness* (lack of awareness of one's negative emotions), *Clarity* (difficultly to discriminate between different negative emotional states), *Strategies* (perception of a lack of available emotion regulation strategies), *Goals* (difficulty to pursue goal-directed behavior when experiencing negative emotional states), and *Impulse* (tendency to act rashly when experiencing a negative emotion). In our study, DERS showed good internal consistency with a Cronbach alpha coefficient reaching 0.90.

#### Depression Anxiety and Stress Scale 21 Items

Depression Anxiety and Stress Scale 21 items (59, 60) is a self-report questionnaire consisting of 21 items on a four-point Likert-type scale. The instrument provides scores for three subscales evaluating levels of depression, anxiety, and stress. In our study, each of these subscales evidenced a good internal consistency with Cronbach's alpha coefficients being 0.91 (*Stress*), 0.88 (*Depression*), and 0.84 (*Anxiety*).

### Statistical Analyses

The statistical analysis plan consisted of several steps. First, descriptive analyses were performed calculating Cronbach's alphas, frequencies, means, and standard deviations of the main variables of the study. Then, the normality of the main variables involved in the study at Time 1 was checked throughout the computation of skewness and kurtosis (61). Normality indexes all fall in the acceptable range except for the Depression Anxiety and Stress Scale Anxiety variable, which showed a normality index slightly upon the acceptable cutoff (Kurtosis = 3.04). Also, bivariate *r*-Pearson correlations were calculated to examine zero-order correlations between continuous variables involved in the study. These analyses were performed with SPSS v.24 for Windows. Then, to test the hypotheses of the study, a structural equation model was designed and tested using the *lavaan* package of the *R* software for Mac. In the models, we inserted age, sex, and education level as control variables on levels of depression, anxiety, and stress measured at Time 2. Also, we added the estimation of the covariances between the levels of depression, anxiety, and stress at Time 1 on the one hand and age, sex, and education level on the other hand. Regarding sex and education variables, they were treated as a dummy variable with 0 being females and 1 being male for sex variable and 0 being having a high school diploma or lower educational level and 1 having a higher educational level for the education variable. The method used evaluates the consistency of a dataset with a model previously defined throughout the robust maximum likelihood method of estimation. Results brought by these statistical analyses are typically judged using several goodness of fit indexes such as root mean square error of approximation (RMSEA), Tucker–Lewis coefficient (TLI), and comparative fit index (CFI). A 0.05 < RMSEA >0.08 (62) and both TLI and CFI being >0.90 (63) are generally interpreted as an adequate fit. In addition, we examined the lower and upper boundaries of the 90% confidence interval for RMSEA, with an upper boundary of more than 0.10, indicating that the model should be rejected (62).

## Results

### Descriptive Analyses

A total of 1,323 respondents answered the survey on Time 1. The age of participants ranged from 18 to 91 years (*M*_*age*_ = 35.38; *SD* = 14.08), and 23% of the sample were male. As for the characteristics of the residence, 49.2% of participants live in a big city, 33.8% in a medium-size city, and 17% in a small city or rural area. Of the participants, 52.3% achieved a high school diploma or a higher level of education. Also, almost half of the sample reported having an income of less than € 36,000 per year, and 26.5% of the participants referred to have one or more children. Because of a COVID-19 emergency, 52.6% reported having to interrupt their working activity. From this first sample, 308 of the respondents (*M*_*age*_ = 35.31; *SD* = 13.91; 22.7% males) participated in the second part of the study. Means and standard deviations were computed for the main variables involved in the study, both for the Total sample (Time 1) and the subsample (Time 2). These are displayed in Table 1.

**Table 1:** Means and Standard Deviations of the main variables involved in the study for the full sample (Time 1) and the subsample (Time 2).

	**Time 1 (*****N*** **=** **1,323)**		**Time 2 (*****n*** **=** **308)**	
	**Mean**	**SD**	**Mean**	**SD**
Age	35.38	14.08	35.31	13.91
UCLA Intimate	24.55	5.84	24.87	5.95
UCLA Social	0.81	1.25	1.07	1.51
UCLA Affiliative	3.35	2.03	3.86	2.35
DERS Total Score	37.97	10.85	37.58	10.85
DASS Depression	5.55	4.35	5.97	4.61
DASS Anxiety	3.01	3.46	2.84	3.41
DASS Stress	7.18	4.80	7.57	4.54

*SD, Standard Deviation; UCLA, UCLA Loneliness Scale; DERS, Difficulties in Emotion Regulation Scale; DASS, Depression Anxiety and Stress Scale*.

### Correlations Between Variables

*r*-Pearson correlations between all variables involved in the study at Time 1 were calculated. Results are fully displayed in Table 2.

**Table 2:** Correlations between main variables of the study.

	**UCLA**	**DERS**	**Anxiety**	**Depression**	**Stress**	**Age**
UCLA	–					
DERS	0.54	–				
Anxiety	0.39	0.45	–			
Depression	0.61	0.63	0.60	–		
Stress	0.45	0.52	0.68	0.70	–	
Age	−0.17	−0.26	−0.23	−0.20	−0.23	–

*UCLA, UCLA loneliness Scale; DERS, Difficulties in Emotion Regulation Scale. All correlation coefficient were statistically significant at p < .001*.

### Structural Equation Model at Time 1

To reach the aims of the study, we tested the goodness of fit of a Structural Equation Model on the whole sample participating at Time 1. Specifically, we tested, controlling for age, sex, and education levels, if UCLA scores predicted depression, anxiety, and stress levels both directly and indirectly throughout emotion dysregulation levels. To create depression, anxiety, and stress latent variables, we used the scores obtained on the items converging on the subscales of Depression Anxiety and Stress Scale-21. Loneliness Latent Variable was produced using the scores obtained on the three subscales (Intimate, Social, and Affiliative) of UCLA. Finally, the emotion dysregulation latent variable was formed using the scores obtained on the six subscales of DERS. Regarding covariates, we observed that age was significantly related to stress, education level with depression, and sex with both anxiety and stress levels.

**Figure 1:**
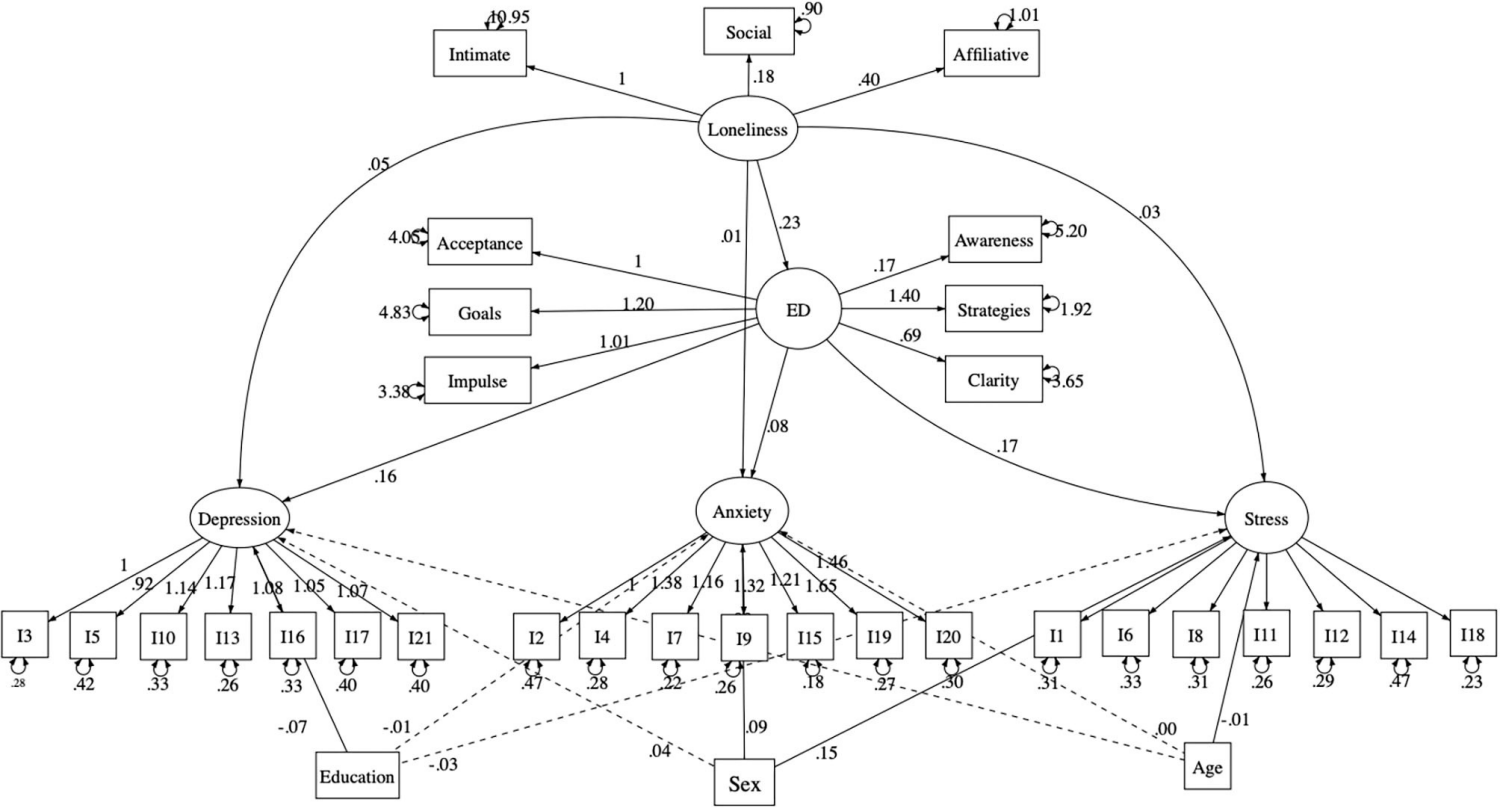
Structural equation model testing the cross-sectional meditational effect of emotion dysregulation in the relationship linking loneliness with anxiety, depression, and stress. Solid lines indicate statistically significant paths. Dashed line indicate not statistically significant paths. ED, Emotion Dysregulation; I, Depression Anxiety Stress Scale item.

As illustrated in Figure 1, the model obtained an acceptable fit as indicated by the CFI (0.91), TLI (0.90), and RMSEA indexes [0.06; confidence interval 90% (0.055–0.060)]. Moreover, all manifest variables loaded significantly on the respective latent variables. Also, we observed that UCLA scores predicted both, directly and indirectly, depression, anxiety, and stress levels.

### Cross Lagged Panel Model on Longitudinal Data

**Figure 2:**
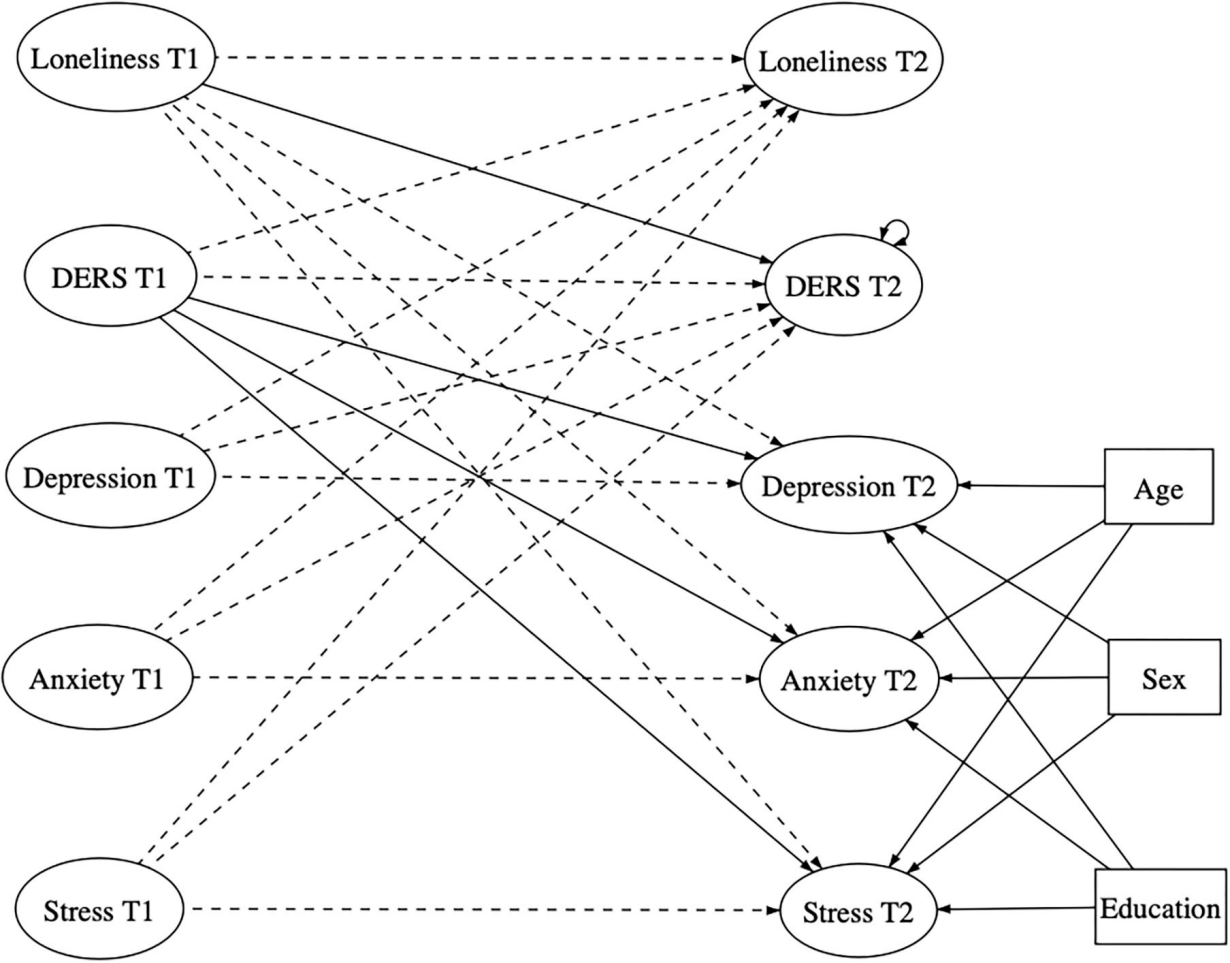
Representation of the full tested longitudinal model. Solid lines indicate statistically hypothesized significant paths. Dashed line indicate paths inserted in the statistical mode but not expected by hypotheses. DERS, Emotion Dysregulation; T1, Scores obtained at Time 1; T2, Scores obtained at Time 2.

To test if emotion dysregulation levels actually mediated the longitudinal link between loneliness and internalizing outcomes (depression, anxiety, and stress), we test a cross-lagged panel model throughout structural equation modeling on the subsample of longitudinal data. The tested paths and the hypothesized model are fully illustrated in Figure 2.

The first model test obtained an adequate fit according to the RMSEA index [0.052; CI 90% (0.049–0.056)] but was slightly below the acceptable CFI cutoff (0.88). The full model is illustrated in Figure 2. Consequently, we consulted the modification indexes and freed some parameters, further estimating some covariance between residual errors of some items involved in the measurement model. To obtain an adequate fit, nine parameters had to been added to the model. All of the additional covariances estimated were between manifest variables converging toward the same latent variable, maintaining coherence with our conceptual model.

**Figure 3:**
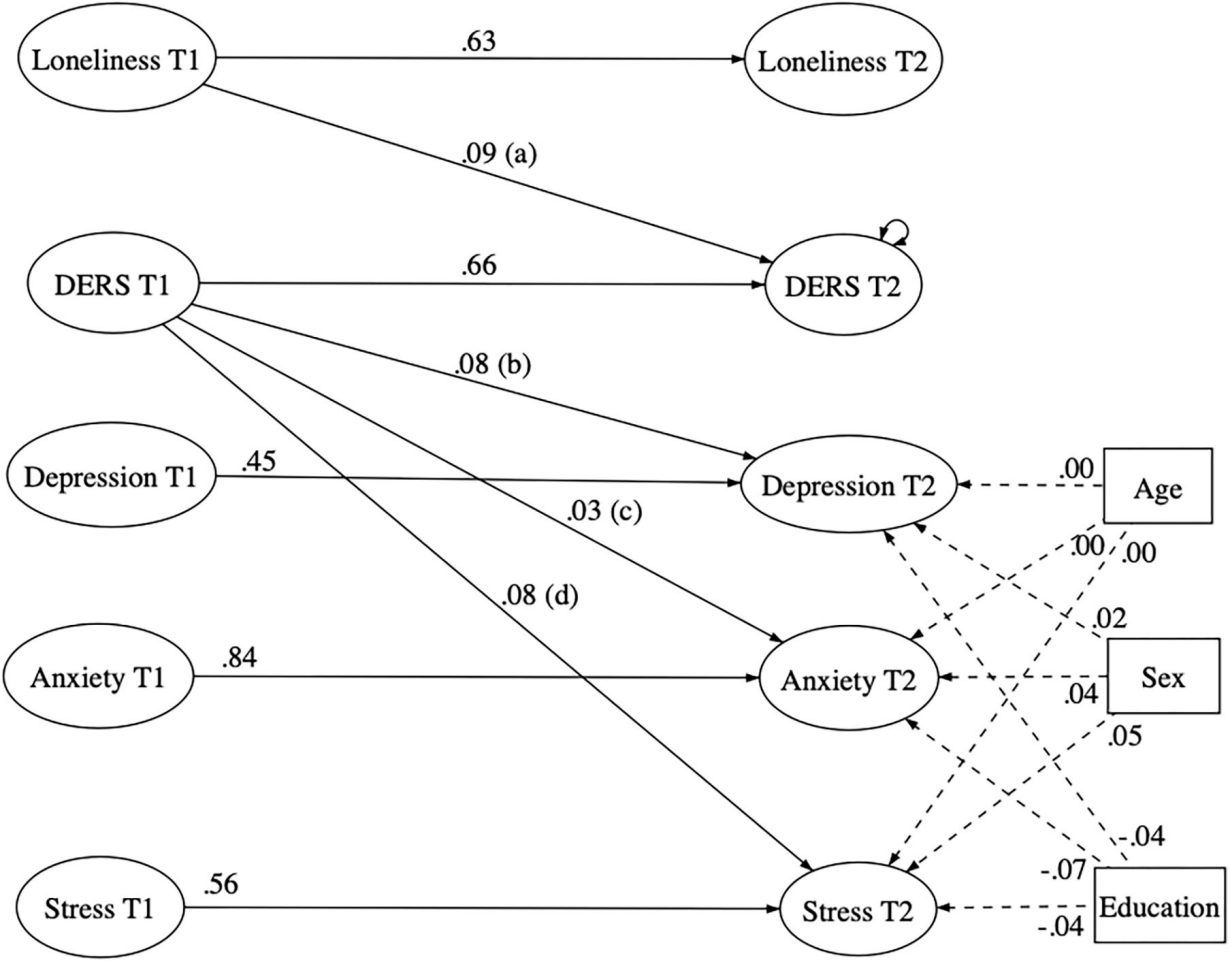
First structural equation model testing the longitudinal meditational effect of emotion dysregulation in the relationship linking loneliness with anxiety, depression, and stress. Solid lines indicate statistically significant paths. Dashed line indicate not statistically significant paths. DERS, Emotion Dysregulation; T1, Scores obtained at Time 1; T2, Scores obtained at Time 2; a*b = 0.01 (*p* = 0.043); a*c < 0.01 (*p* = 0.061); a*d = 0.01 (*p* = 0.017).

The final model [RMSEA = 0.50; CI 90% (0.046–0.053); CFI = 0.90] is illustrated in Figure 3. Directions and statistical significances of coefficients did not differ from the first model tested. For clarity purposes, we did not display coefficients between manifest and latent variables, but all of them were positive and significant. Moreover, we found that emotion dysregulation levels partially mediated the longitudinal relationship between loneliness and both depression (β = 0.006; *p* = 0.043) and stress (β = 0.007; *p* = 0.017) but not between loneliness and anxiety levels (β = 0.002; *p* = 0.061).

## Discussion

It is widely stated that in times of crisis, psychological research should address and provide answers on risk and protective factors involved in the well-being and adjustment of individuals. Empirical and anecdotal contributions have highlighted that the emergency related to COVID-19 has been very powerful in physical and psychological impacts. In the wide range of sudden changes undergone by the world population, most of them have had to face restrictive measures related to their interpersonal interactions: in many countries, the population has been asked to maintain rigorous and lasting lockdown measures to contain the pandemic. One of the consequences for most people was a reduced frequency of social interactions, which was probably translated into an increase in the level of loneliness perceived for most of them. Our focus on loneliness is linked to results that suggest that one of the most direct consequences of lockdown measures is isolation and the associated feeling of loneliness (64). Because previous research has already highlighted the negative consequences of loneliness on mental health, particularly on the maintenance, worsening or even onset of internalizing symptoms, clinical psychologists have been asked to address this central issue during the COVID-19 emergency.

However, the available empirical evidence that can provide useful clinical indications for dealing with psychological problems caused by “isolation” is limited. Our study aimed to test the hypotheses that an increase in loneliness levels would predict an increase in internalizing symptom levels and that the ability to regulate emotions may mediate these relationships. Our focus on emotion regulation strategies as a further mediator of these associations has its roots in the widely coherent literature that attests to their association with psychopathology and, more specifically, with reactions to distressing events and situations (65, 66).

The first equation structural model was based on an evaluation of all the variables at Time 1 when 1,323 participants were at the beginning of the lockdown. In this model, a significant relationship has been observed between the dimensions of loneliness, emotional dysregulation, and internalizing symptoms. Specifically, loneliness dimensions directly predicted depression, anxiety, and stress levels. This datum is in line with existing literature concerning the association between loneliness and anxiety, depression and other psychological outcomes, and even suicide (25, 67). However, if the majority of past studies focused their attention on chronic loneliness (68), in this study, we can start a reflection on the similar impact of intense and constrictive isolation.

Furthermore, we found that loneliness predicts these results in terms of depression, anxiety, and stress levels also indirectly, mediated by emotion regulation strategies. In fact, solitude, in its aspects of the intimate, social, and affiliative components, has its most distressing effect if associated with difficulties in emotion regulation. Other studies have shown this strong association (69) and have investigated the role of emotion regulation strategies as a mediator of the sense loneliness perceived (70), whereas other authors have investigated loneliness itself as a mediator in the association between emotion regulation strategies and psychological outcomes (71), showing that loneliness mediates the association between the difficulties in regulating emotions and psychopathology.

The second equation structural model aimed at testing the role of time in a subsample of 308 participants concerning these associations. The results of this model only partially supported what was found in the cross-sectional analysis: the levels of emotion dysregulation mediated only the path that connects perceived loneliness and depression and stress but not anxiety. Regarding anxiety, we may speculate that other factors may explain the longitudinal relationship between loneliness and anxiety levels. For instance, the lack of social confrontation on the topic generating anxiety (such as news related to the pandemic) and the fear of getting sick in a situation of loneliness may better explain the pathways linking perceived loneliness to anxiety. An additional explanation of this result can be found in methodological considerations. Indeed, the important dropout of participants between Time 1 and Time 2 of the study has induced an important loss of statistical power, thus making not significant a precedent significant path. We may speculate so that a bigger sample size at Time 2 (almost equal to those used at Time 1) would have evidenced a significant influence of emotion dysregulation in the path between perceived loneliness and anxiety, in line with the high number of studies highlighting the role played by this variable in anxiety symptomatology.

Regarding the role of emotional dysregulation in the relationship between loneliness and stress levels, several considerations can be made. First, our results are in line with previous literature showing that adaptive emotion regulation strategies, such as reappraisal, may buffer the negative impacts of daily stress on positive emotions (72) and that emotion dysregulation and coping stress seem to share a common neurobiological basis (73) underlying the tight relationship between emotion regulation capacities and stress resilience. Moreover, medical literature documented the association between loneliness status and stress, operationalized throughout physiological variables, including indicators such as stress hormones (74). Indeed, researchers brought relevant pieces of evidence highlighting the central role of social support in resilience to stress (75). In the context of the pandemic and confinement, we can hypothesize that loneliness may have deprived individuals of basic emotion regulation strategies such as exercise (76) and, in turn, account for increased levels of excessive arousal (e.g., fatigue). Similarly, lack of perceived social support associated with loneliness would probably heighten the feeling of being more easily overwhelmed by external threats, such as health problems, job loss, or domestic violence (77, 78). Besides, loneliness may interrupt the buffering role of social interactions toward maladaptive rumination that would, in turn, lead to increased levels of stress (79). Thus, owning good emotion regulation capacities is likely to play a protective role in the pathways leading loneliness to stress, balancing the deprivation of interpersonal protective factors in relation to stress.

Regarding the mediating role of emotion dysregulation in the relationship between loneliness and depression, several explanations can be provided. For instance, a difficulty to accept negative emotions triggered by loneliness feelings in a non-judgmental way may lead to depressive symptoms, eliciting thoughts on the self-perceived inability to adequately face the situation. In this regard, adequate information provided by the institutions on the potential negative emotional states aroused by the situation can be of great use in legitimizing the onset of this emotion in the entire population. Furthermore, an alleged lack of self-efficacy in regulating emotions can be reasonably explained by our observations. This may be especially true of individuals with low self-efficacy in regulating emotions, who have perceived themselves as accustomed to relying on others to regulate their negative emotional states. Feeling lonely for individuals with a propensity for this emotional regulation deficit can be particularly inspiring. In this regard, a prevention program that aims to provide some tips for regulating negative emotions can help avoid a major increase in psychological distress in the general population. Furthermore, from this perspective of understanding the associations between vulnerability factors and psychological well-being, other interesting discoveries offer ideas for further research on these topics. For example, an interesting fact concerns the role of age in the mediation of these associations. In fact, we have found significant correlations between age and outcomes in terms of depression, anxiety, and stress. Young people show multiple levels of stress during this blocking period. It can be suggested that abandoning important aspects of their daily lives for young people could be more stressful.

Other studies (80, 81) have pointed out that the closure of schools, universities, and businesses has led to negative feelings and has greatly impacted the population in terms of mental health. We could suggest that the closure of the productive sectors mentioned and also of other important sectors, e.g., cultural and sports centers and other recreational activities, have a greater impact on young people who have greater difficulty in abandoning their previous lifestyle. However, this preliminary data need further sociodemographic insights to be better explained.

Despite the insightful nature of our findings, several limitations of the study need to be considered. First, despite longitudinal studies often suffer from relevant dropout in the participation of subjects, this issue is likely to introduce a bias in our study. Indeed, we are not aware of the reasons for dropping, and dropped participants may be more resilient or, conversely, more vulnerable to some of the mental health issues investigated in our study. This issue may limit the generalizability of our findings. Then, due to the growing research on COVID-19, we have chosen to focus attention on the general population, and we have not been able to define specific reactions between subsamples of more exposed and less exposed people. However, our idea is that it is equally important that research may focus on the most influential risk factors. In this line, as suggested by other authors (80), the possibility of capturing psychological responses to the pandemic represents itself a crucial element in identifying people who may need psychological intervention.

## Conclusion

Overall, our results indicate the need to improve research in defining specific risk factors implicated in well-being and adjustment to this very demanding period. This could suggest implementing intervention programs aimed at improving well-being in population segments most at risk for their functioning and mental health.

## Data Availability Statement

The raw data supporting the conclusions of this article will be made available by the authors, without undue reservation.

## Ethics Statement

The studies involving human participants were reviewed and approved by Sapienza Ethics Board. The patients/participants provided their written informed consent to participate in this study.

## Author Contributions

PV and RT conceived the presented idea. SB, RC, and GR performed the analysis. All authors discussed the results and contributed to the final manuscript.

## Conflict of Interest

The authors declare that the research was conducted in the absence of any commercial or financial relationships that could be construed as a potential conflict of interest.
